# Three-Dimensional Shape Measurement Using Speckle-Assisted Phase-Order Lines Without Phase Unwrapping

**DOI:** 10.3390/s26082534

**Published:** 2026-04-20

**Authors:** Ziyou Zhang, Weipeng Yang

**Affiliations:** School of Artificial Intelligence, Chengdu Technological University, Chengdu 611730, China; zzyou1@cdtu.edu.cn

**Keywords:** 3D shape measurement, fringe projection, speckle projection, phase-order line, stereo matching, structured light

## Abstract

Achieving high-accuracy and high-speed 3D shape measurement remains a significant challenge. This paper presents a novel technique using phase-order lines (POLs), which eliminates the need for phase unwrapping in a binocular system. By combining phase-shifting for high resolution and speckle projection for robust features, our method extracts POLs directly from the wrapped phase. The speckle patterns are then used to establish robust POL correspondences between stereo images. These matched POLs serve as reliable seeds to guide dense, sub-pixel matching directly on the wrapped phase, thus bypassing the complex phase unwrapping process. This approach significantly reduces the number of required patterns. The experimental results demonstrate that our method achieves a root-mean-square (RMS) error of 0.058 mm using only five patterns, delivering accuracy comparable to a 12-pattern temporal phase unwrapping (TPU) method while being significantly faster.

## 1. Introduction

High-resolution, real-time three-dimensional (3D) shape measurement is crucial for applications such as industrial inspection [1,2,3], reverse engineering [4], biomedical imaging [5], cultural heritage digitization [6], and entertainment [7]. Optical 3D measurement techniques are broadly classified into passive and active approaches [8]. Passive stereo vision reconstructs geometry from an object’s natural texture using stereo matching algorithms [9,10]. However, its accuracy is limited to textureless surfaces, and achieving dense matching is computationally expensive.

Active methods overcome these limitations by projecting pre-defined patterns onto an object’s surface. This category of techniques is broadly known as structured light. It is important to distinguish this term from its use in fundamental optics, where “structured light” often refers to the sophisticated spatial engineering of a light beam’s phase and polarization to generate complex modes like optical vortices [11,12]. In the context of this paper and the broader field of 3D metrology, structured light specifically refers to projecting intensity patterns to encode surface geometry. Among these projection techniques, fringe projection profilometry (FPP) [13,14,15,16,17] and speckle projection [18,19,20] are widely used. FPP encodes the surface with a continuous phase, commonly retrieved using phase-shifting profilometry (PSP) [13,14] or Fourier transform profilometry (FTP) [15,16]. PSP is generally more accurate and robust [17]. Nevertheless, the calculated phase is wrapped within the range of (−π, π] and requires phase unwrapping.

Phase unwrapping methods include spatial phase unwrapping (SPU) [21,22,23] and temporal phase unwrapping (TPU) [24,25,26] approaches. SPU algorithms analyze the wrapped phase map itself but are prone to error propagation and fail on spatially isolated regions (“islands”) [21]. TPU methods project additional fringe patterns at different frequencies to resolve ambiguity at each pixel independently. While they offer high reliability, this advantage comes at the cost of increased acquisition time, making them sensitive to motion [24,25,26].

Speckle-based methods project random patterns and use digital image correlation (DIC) for stereo matching [18,19,20,27]. While effective for textureless objects, the spatial resolution of these methods is constrained by the correlation window size and the projector resolution [8].

To leverage the complementary strengths of both techniques, combined approaches have been developed. Some methods use speckle for coarse depth estimation and phase for refinement [28], while others embed speckle-like signals directly into sinusoidal fringes [29,30,31]. However, this embedding process can degrade fringe quality [30,31]. Recently, deep learning has been applied for absolute phase recovery [32,33] and stereo matching [34], but these methods often require large datasets and complex network architectures. Table 1 provides a comparison of these representative methods.

We propose a speckle-assisted POL method that differs from prior art as follows: it avoids global phase unwrapping by detecting phase transition points (PTPs) in the wrapped phase and linking them into POLs; these POLs are matched across views using a single binary speckle image to obtain unique seed correspondences, followed by narrow-window refinement directly in the wrapped phase to produce dense, subpixel matches and avoid island errors. Compared with TPU, it requires only five projections; unlike DIC, speckle is used only for skeleton matching, while sinusoidal phase delivers subpixel accuracy; unlike speckle-embedded fringes, speckle and fringes are projected separately to preserve phase quality; and unlike learning-based methods, no training is required.

Main contributions:

1. POLs: continuous curves extracted from the wrapped phase; their speckle-based matching yields robust initial correspondences.
2. A two-stage pipeline (coarse POL matching + fine wrapped-phase refinement) requiring only five patterns to obtain dense disparity.
3. Intrinsic robustness to spatially isolated objects: independently matched POLs circumvent SPU’s island problem and approach TPU-level reliability with fewer projections.

## 2. Materials and Methods

### 2.1. System Configuration

**Figure 1:**
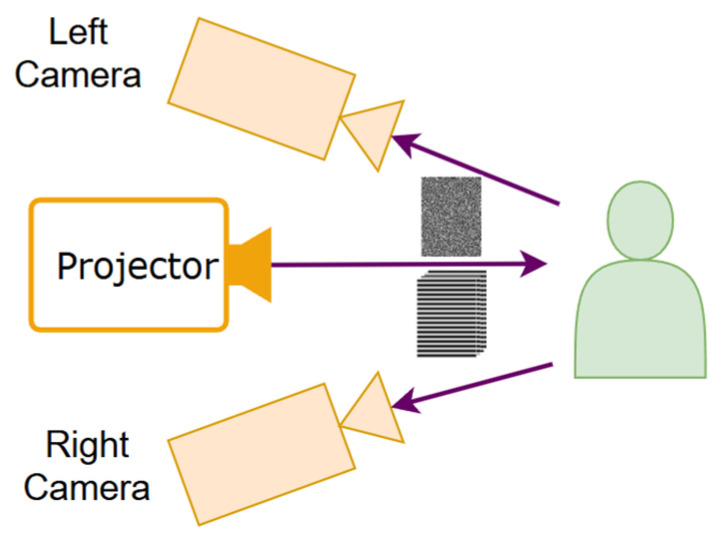
Illustration of the binocular structured light system.

The proposed measurement system adopts a binocular stereo vision configuration with a digital light processing (DLP) projector placed between two cameras, as illustrated in Figure 1. The projector sequentially projects four phase-shifted sinusoidal fringe patterns and one binary speckle pattern onto the measured object. The two cameras capture the corresponding images synchronously. The system is calibrated using Zhang’s flexible calibration method [35], and all captured images are rectified such that the epipolar lines are aligned horizontally, enabling efficient one-dimensional disparity search.

**Figure 2:**
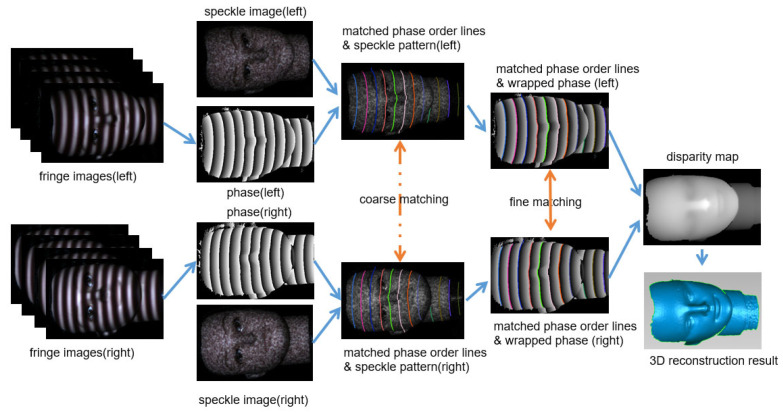
Framework of proposed 3D reconstruction method. Stereo matching proceeds in two stages: speckle-based POL pairing, followed by wrapped-phase refinement for dense sub-pixel disparity. POLS of the same color in the left and right images represent matched POLS with the same index.

The algorithm framework is presented in Figure 2.

### 2.2. Four-Step Phase-Shifting Algorithm

**Figure 3:**
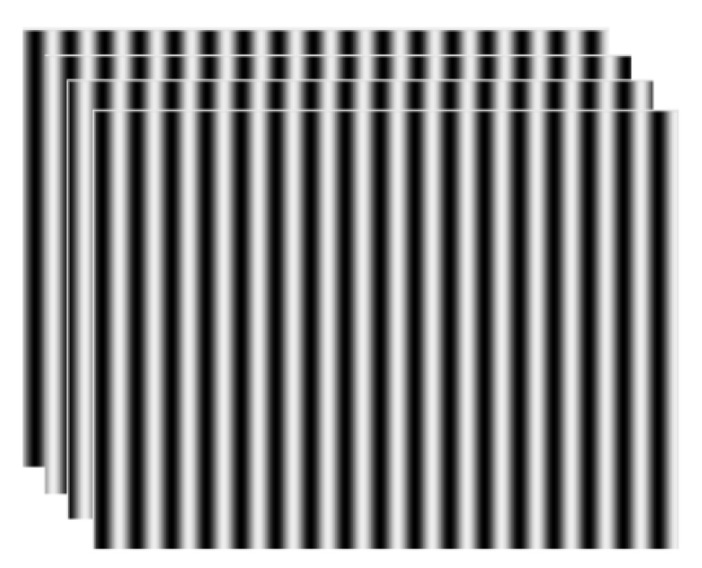
Sinusoidal fringe patterns.

PSP is widely used in optical 3D measurement due to its high accuracy and robustness to noise [13,14,17]. In the four-step phase-shifting algorithm, four sinusoidal fringe patterns with a phase shift of π/2 (Figure 3) are sequentially projected. The intensity captured at pixel (x, y) for the n-th pattern is modeled as:

(1) $$I_{n}\left(x,y\right)=I_{b}\left(x,y\right)+I_{m}\left(x,y\right)\cos\left[\varphi \left(x,y\right)+\frac{2\pi n}{N}\right],n=0,1,2,3$$

where $I_{b}\left(x,y\right)$ is the background intensity, ${\;I}_{m}\left(x,y\right)$ is the modulation intensity, and $\varphi \left(x,y\right)$ is the phase to be recovered. The wrapped phase is computed as:

(2) $$\varphi \left(x,y\right)=\arctan\left[\frac{I_{3}\left(x,y\right)-I_{1}\left(x,y\right)}{I_{0}\left(x,y\right)-I_{2}\left(x,y\right)}\right]$$

Due to the arctangent operation, the computed phase $\varphi \left(x,y\right)$ is wrapped in the interval of (−π, π]. The modulation intensity, which serves as a reliable indicator of data validity, is calculated as:

(3) $$I_{m}\left(x,y\right)=\frac{1}{2}\sqrt{[I_{0}(x,y)-I_{2}(x,y){]}^{2}+[I_{1}(x,y)-I_{3}(x,y){]}^{2}}$$

Pixels with a modulation intensity below a predefined threshold are considered unreliable and thus excluded from subsequent processing.

### 2.3. Phase Order Line

The relationship between the absolute phase ϕ(x,y) and the wrapped phase *φ*(*x*, *y*) is given by:

(4) $$\phi \left(x,y\right)=\varphi (x,y)+2\pi k(x,y)$$

where k(x,y) is an integer field known as the phase order. The phase order can be formally defined as:

(5) k(x,y)=round(ϕ(x,y)−φ(x,y))/2π

where round() denotes the rounding to the nearest integer. The value of *k*(*x*, *y*) changes only at locations where the wrapped phase *φ*(*x*, *y*) exhibits a 2π discontinuity. These discontinuity locations form continuous curves, which we term POLs.

(6) $$I_{\mathrm{jump}}\left(x,y\right)=\left\{\begin{array}{c} 0,\;\;\;\left|\varphi \left(x+1,y\right)-\varphi \left(x,y\right)\right|<T\_\phi \\ 1,\;\;\;\left|\varphi \left(x+1,y\right)-\varphi \left(x,y\right)\right|\geq T\_\phi \end{array}\right.$$

Instead of performing full phase unwrapping to compute *k*(*x*, *y*), our method directly detects these discontinuities. We define a Phase Transition Point (PTP) as a pixel where the phase difference between adjacent horizontal pixels exceeds a threshold. The PTP map *I*_jump_(*x*, *y*) is calculated as:

The threshold T_ϕ is set to 1.5π. This choice is critical for robustly distinguishing true phase wraps from phase variations caused by steep surface gradients. A value of 1.5π provides a large safety margin, as any phase gradient steep enough to exceed this threshold between adjacent pixels would likely violate the sampling theorem, leading to phase ambiguity. The method is not highly sensitive to this parameter; our empirical tests show stable performance for T_φ in the range of [1.2π, 1.8π]. A lower threshold risks misidentifying steep surfaces as wraps (false positives), while a higher threshold may fail to detect noisy phase wraps (false negatives).

**Figure 4:**
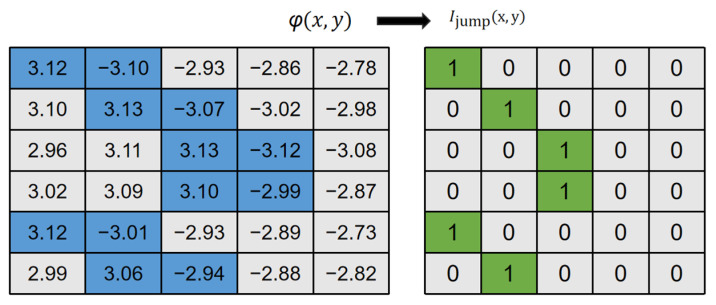
Schematic diagram of calculating PTP. The blue grids represent the phase value near the PTP, and the green grids represent the PTPs.

The schematic diagram of PTP extraction is shown in Figure 4.

**Figure 5:**
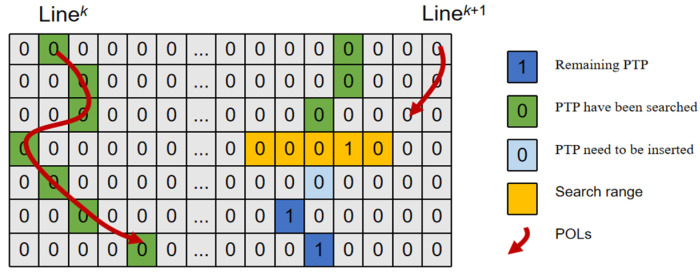
Schematic of the iterative search strategy for extracting a continuous POL. The grid represents the PTP map $I_{\mathrm{jump}}$. The search for a new POL (red arrow, Line^(*k*+1)^) starts from an unsearched PTP (blue square, “Remaining PTP”). The algorithm iteratively finds the next point within a local search range (yellow squares) in the +*y* direction. PTPs that have already been assigned to a previous POL (Line*^k^*) are marked as “searched” (green squares) and are ignored. If a gap is detected (e.g., due to noise), a point is inserted via linear interpolation (light blue square, “PTP needs to be inserted”) to maintain the continuity of the line.

Continuous POLs are extracted by sequentially connecting PTPs in the $I_{\mathrm{jump}}$ map. The search algorithm proceeds along the +*y* direction, linking adjacent PTPs within a local neighborhood, as illustrated in Figure 5. To ensure continuity, any gaps in the POLs caused by noise or occlusions are filled using linear interpolation. The resulting sets of POLs for the left and right views, denoted as $Lin{eVec}_{L}$ and $Lin{eVec}_{R}$, are then ready for the coarse matching process.

### 2.4. Binary Speckle Pattern

The binary speckle pattern is generated using the method described by Schaffer et al. [36]. A random matrix of the same resolution as the projector (1280 × 800 pixels) is created, where each pixel is independently assigned a value of 0 (black) or 255 (white) with equal probability. To ensure that the projected speckle has sufficient feature size for reliable correlation, a minimum speckle size of 3 × 3 pixels is enforced by applying morphological dilation. The resulting binary pattern is stored as a bitmap and loaded into the DLP projector.

**Figure 6:**
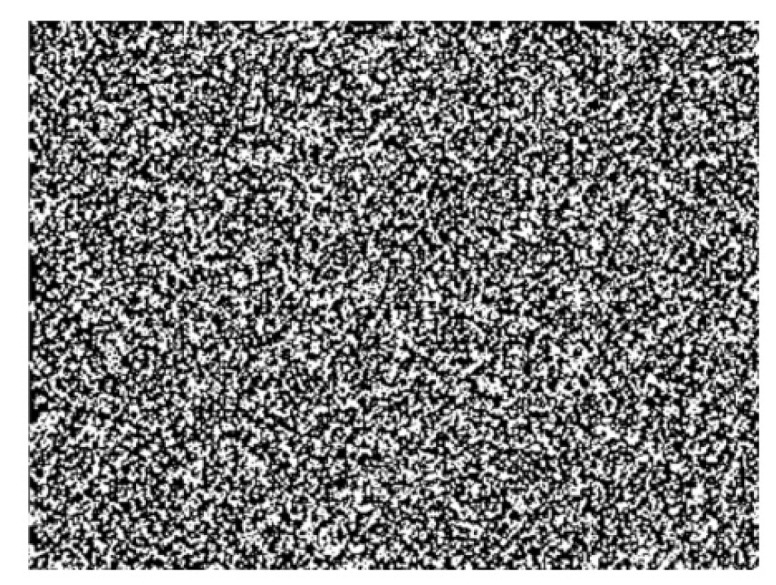
Speckle pattern (cropped).

Binary speckle patterns offer several advantages over grayscale speckle patterns for the proposed application [29,37]: (1) binary patterns can be projected at higher speed by DLP projectors operating in binary mode; (2) they produce higher contrast on the object surface, thereby improving the signal-to-noise ratio; and (3) their unique local intensity distribution ensures robust template matching. The projected speckle pattern (cropped) is shown in Figure 6.

### 2.5. Stereo Matching

This section details a two-stage stereo matching methodology, comprising a coarse matching stage to establish initial correspondences, followed by a fine matching stage for dense, sub-pixel disparity refinement.

#### 2.5.1. Similarity Metric of Speckle

To robustly quantify the matching similarity between corresponding pixel regions, the Zero-Mean Normalized Cross-Correlation (ZNCC) [38] metric is employed. ZNCC is highly resilient to linear variations in brightness and contrast.

(7) $$C_{ZNCC}\left(x,y,u,v\right)=\frac{\sum_{i=-M}^{M}\sum_{j=-M}^{M}\left[I_{L}\left(x+i,y+j\right)-\bar{I_{L}\left(x,y\right)}\right]\;\left[I_{R}\left(u+i,v+j\right)-\bar{I_{R}\left(u,v\right)}\right]}{\sqrt{\sum_{i=-M}^{M}{\sum_{j=-M}^{M}\left[I_{L}\left(x+i,y+j\right)-\bar{I_{L}\left(x,y\right)}\right]}^{2}\sum_{i=-M}^{M}\sum_{j=-M}^{M}{\left[I_{R}\left(u+i,v+j\right)-\bar{I_{R}\left(u,v\right)}\right]}^{2}}}$$

where $I_{L}\left(x+i,y+j\right)$ represents the pixel intensities of the left image, and $I_{R}\left(u+i,v+j\right)$ denotes the pixel intensities of the right image. $\bar{I_{L}\left(x,y\right)}$ and $\bar{I_{R}\left(u,v\right)}$ represent the mean intensities within an (2*M* + 1) × (2*M* + 1) window. ZNCC represents the window size. Larger windows improve robustness in low SNR but may blur thin structures and introduce bias across depth discontinuities; smaller windows preserve detail but are more noise-sensitive. In our tests, varying the window from 7 × 7 to 13 × 13 changed seed density by <1% and the final disparity RMSE by <0.02 pixels. The similarity value of $C_{ZNCC}\left(x,y,u,v\right)$ lies in the range from −1 to 1; the larger the value, the higher the similarity.

The parameters for speckle-based matching, including the speckle size (3 × 3 pixels), the use of a high-contrast binary pattern, and the ZNCC correlation window size (9 × 9 pixels), were selected based on established practices in the digital image correlation and stereo matching literature [39], and were empirically confirmed to be effective for our system.

#### 2.5.2. Coarse Matching of POL Pairs

**Figure 7:**
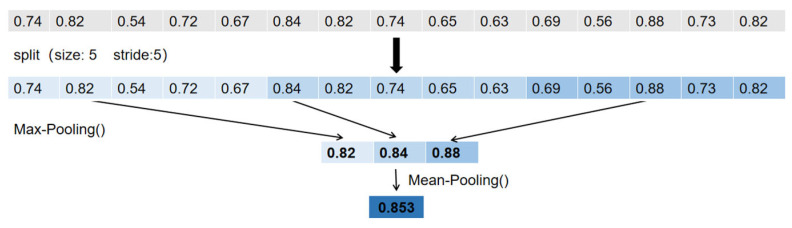
Illustration of the robust similarity score calculation for a POL pair. The process begins with a vector of ZNCC similarity scores (top row), where each value represents the similarity of a single PTP pair. (1) Partitioning: The vector is split into local groups (in this example, with a window size and stride of 5). (2) Max-Pooling: The maximum value is selected from each group (e.g., 0.82 is the max of the first group, 0.84 for the second, etc.). This step ensures that the contribution from each local segment is represented by its best-matched feature, thus mitigating the influence of individual low-score outliers. (3) Mean Calculation: The final robust similarity score S (0.853 in this case) is computed by averaging the maximum values obtained from the pooling step. This aggregated score provides a stable and reliable measure of similarity for the entire POL pair.

**Figure 8:**
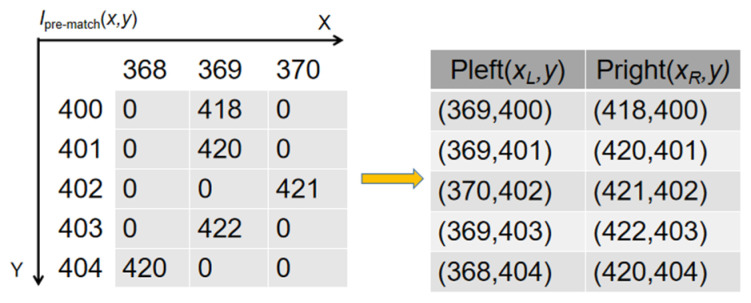
Schematic illustrating the storage and interpretation of coarse matching results. The matched PTP correspondences are stored in a sparse 2D map $I_{pre-match}$. The coordinates (xL, y) of this map correspond to a PTP in the left image, and the value stored at that location is the horizontal coordinate xR of its corresponding PTP in the right image. For example, at position (369, 400), the stored value is 418. This indicates that the left-image point Pleft (369, 400) is matched with the right-image point Pright (418, 400). These sparse point pairs (Pleft, Pright) serve as reliable seed points to guide the dense, fine matching process on the wrapped phase map.

This stage establishes initial, sparse correspondences between candidate POL pairs. For each candidate pair (*L_m_*, *R_n_*), a similarity vector v is first generated by calculating the ZNCC scores for all corresponding PTPs using the speckle images. To mitigate the impact of outliers, a robust similarity score $S\left(L_{m},R_{n}\right)$ is then derived from v through a two-stage aggregation process: a max-pooling operation followed by averaging, as defined in Equations (8) and (9) and illustrated in Figure 7. Finally, for each left-view POL *L_m_*, the right-view POL *R_n_* that yields the highest score *S* is selected as the match, provided this score exceeds a predefined threshold S_th (e.g., 0.5). The resulting matched POL pairs provide a sparse set of reliable seed points for the subsequent fine matching stage. The correspondences of the matched PTPs are stored in a sparse map $I_{pre-match}$, which serves as a set of reliable seed points for the subsequent fine matching stage, as detailed in Figure 8.

(8) $$v^{\prime }=\mathrm{MaxPool}\left(v,s\right)$$

(9) $$S\left(L_{m},R_{n}\right)=mean\left(v^{\prime }\right)$$

#### 2.5.3. Fine Matching on the Wrapped Phase

**Figure 9:**
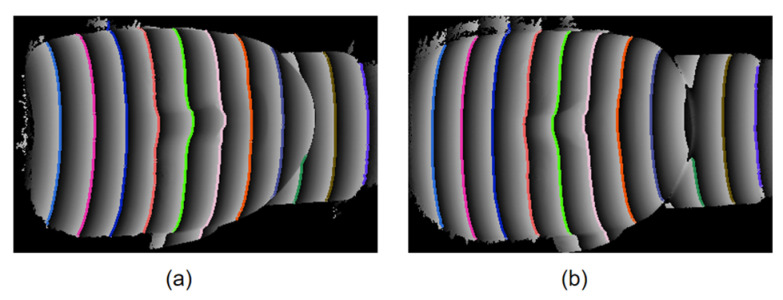
Matched POLs on the wrapped phase. (**a**) Left view. (**b**) Right view. POLS of the same color represent successfully matched left–right POLS.

We performed dense, sub-pixel correspondence directly on the wrapped phase by growing from high-confidence seeds obtained from the matched POL pairs (as shown in Figure 9); same-colored POLs guide propagation and prevent 2π slips.

Step 1 (Seeds): From matched POL pairs, we form reliable left–right seeds and compute their initial disparities, retaining only seeds that pass modulation/polarity checks [37,38].

Step 2 (Dense propagation): Using a flood-fill along scanlines under a narrow search window centered at a disparity predicted from neighboring seeds, we select the phase order *k* that minimizes $\left|\varphi_{L}-{(\varphi }_{R}+2\pi k)\right|$ and restrict matching accordingly [38]. Let *r* and *r* + 1 be adjacent right-image pixels in the narrow window such that $\varphi_{R}(r,y)\leq \varphi_{L}(xL,y)\leq \varphi_{R}(r+1,y)$. With a first-order approximation of phase versus pixel position, the sub-pixel right coordinate is

(10) $${xR}^{*}=r+\left[\varphi_{L}(xL,y)-\varphi_{R}(r,y)\right]/\left[\varphi_{R}(r+1,y)-\varphi_{R}(r,y)\right]$$

and the disparity is $d(xL,y)=xL-{xR}^{*}$.

## 3. Experiment and Results

### 3.1. The Experimental Platform

**Figure 10:**
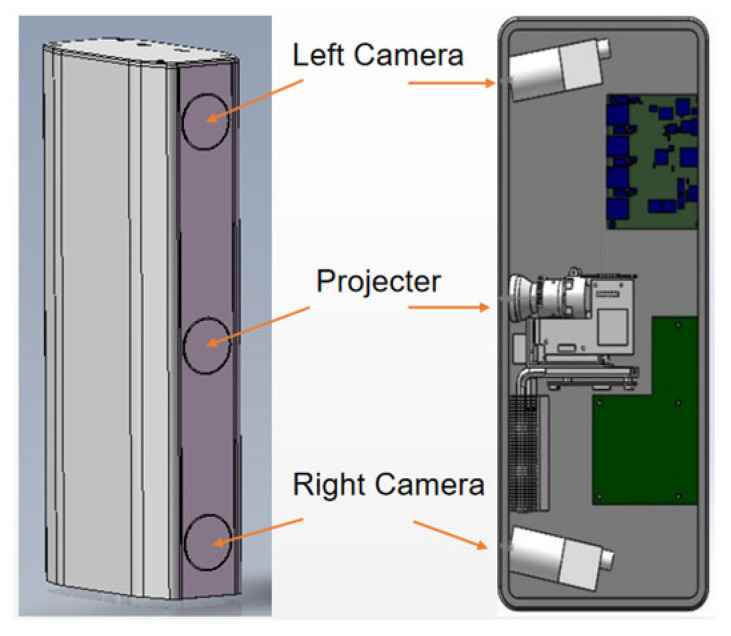
Experimental setup for stereo structured-light measurement. The system consists of two CMOS cameras (daA1920-160uc, Basler AG, Ahrensburg, Germany; 1920 × 1200 px, 160 fps, 5.86 μm pixel size) and a custom-built DLP projector (internal model OPD30, Wisesoft Co., Ltd., Chengdu, China; 1280 × 800 px) placed between the cameras. The stereo baseline is about 300 mm, and the working distance is about 800 mm. Under this configuration, the lateral field of view is approximately 400 × 300 mm, and the theoretical depth resolution is about 0.03 mm. All measurements in Section 3.2 and Section 3.3 used this hardware and geometry unless otherwise noted. The capture sequence for the proposed method comprises five frames (one speckle pattern plus four phase-shifted fringe patterns).

The experimental system is shown in Figure 10.

The projector is equipped with a hardware trigger that drives both cameras to capture images synchronously. A complete measurement cycle consists of projecting five patterns (four phase-shifted sinusoidal fringes at the same frequency with phase shifts of 0, π/2, π, and 3π/2, along with one binary speckle pattern) and capturing the corresponding five image pairs. The fringe frequency is set to 12 periods across the projector width (1280 pixels), corresponding to approximately 107 pixels per period.

The binocular system is calibrated using Zhang’s method [35] with a planar checkerboard target. The calibration provides intrinsic parameters (focal length, principal point, distortion coefficients) and extrinsic parameters (rotation and translation) for both cameras. Image rectification is performed to align the epipolar lines horizontally.

### 3.2. Qualitative Results

For visualization purposes, the captured images were cropped to a 1280 × 1024 region of interest (ROI) and rotated $90^{{}^{\circ}}$ clockwise for display in the figures.

#### 3.2.1. POL Matching Results

**Figure 11:**
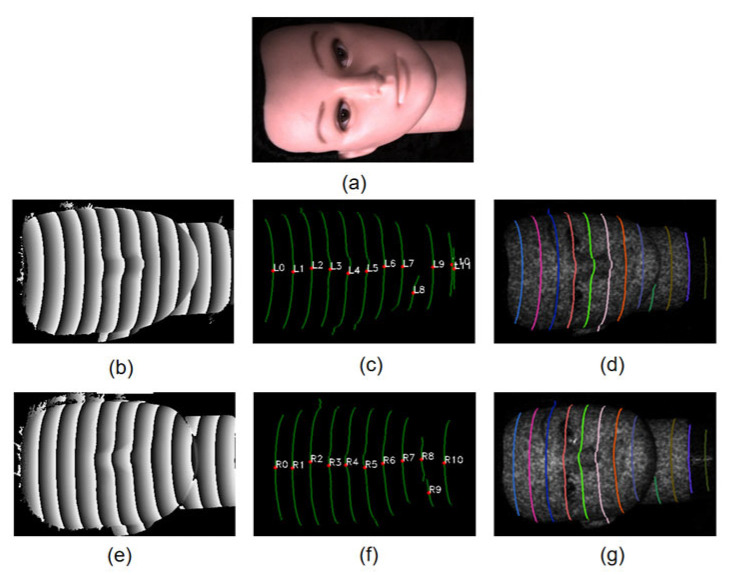
Experiment result of 3D face model. (**a**) Photograph. (**b**) Left wrapped phase map. (**c**) Left POL map. (**d**) Matched POLs on the left speckle image. (**e**) Right wrapped phase map. (**f**) Right POL map. (**g**) Matched POLs on the right speckle image.

A 3D face model was used to demonstrate the POL extraction and matching process. The results are shown in Figure 11. The wrapped phase maps are computed from the four phase-shifted fringe images using Equation (2). The PTP maps and POLs are extracted as described in Section 2.3.

Table 2 shows the ZNCC similarity values between L_1_ and all right-view POLs (R_0_–R_10_). The correct match (R_1_) has the highest similarity score of 0.86, which is well separated from the second highest value of 0.23. Table 3 shows the matching results for all left-view POLs. All POL pairs within the shared field of view are matched correctly. L_10_ and L_11_ have no valid match because they fall outside the overlapping region of the two views.

#### 3.2.2. 3D Shape Measurement of Face Model

**Figure 12:**
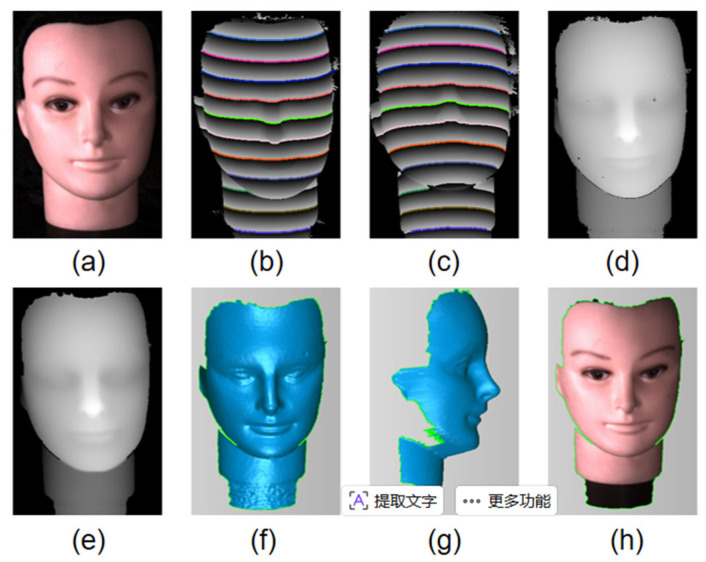
3D reconstruction of the face model. (**a**) Photograph. (**b**,**c**) Wrapped phase and matched POL pairs. (**d**) Disparity map. (**e**) Disparity map after hole filling. (**f**,**g**) Reconstruction result. (**h**) Textured reconstruction.

Figure 12 shows the complete measurement results for the 3D face model. The disparity map (Figure 12d) is obtained through the two-stage matching algorithm. After hole filling using interpolation, this dense disparity map (Figure 12e) is converted to a 3D point cloud via triangulation using the calibrated camera parameters. The reconstructed 3D shape (Figure 12f,h) accurately captures the geometric details of the face, including the nose ridge, eye sockets, and lip contours.

#### 3.2.3. Real Human Face Measurement

**Figure 13:**
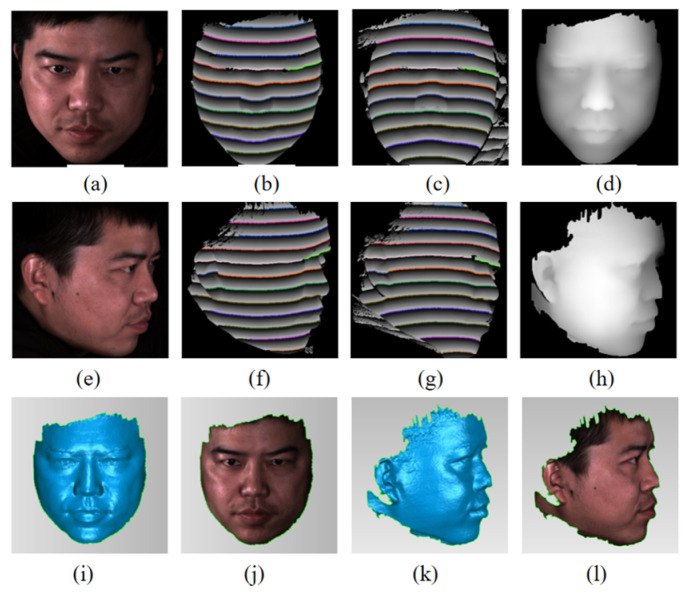
3D reconstruction of real human faces. (**a**,**e**) Photographs. (**b**,**c**,**f**,**g**) Wrapped phase with matched POLs. (**d**,**h**) Disparity maps. (**i**,**k**) Reconstruction result. (**j**,**l**) Textured reconstructions.

Figure 13 shows the measurement results for a real human face in two different poses (frontal and lateral). The results demonstrate that POLs can be successfully matched regardless of pose. The reconstructed surfaces accurately capture facial features, including the ears, nose, and chin. Some areas with dark textures (e.g., hair) or steep surface angles (e.g., facial contour edges) show data loss in these areas, which is attributable to low fringe modulation.

#### 3.2.4. Multiple Isolated Objects

**Figure 14:**
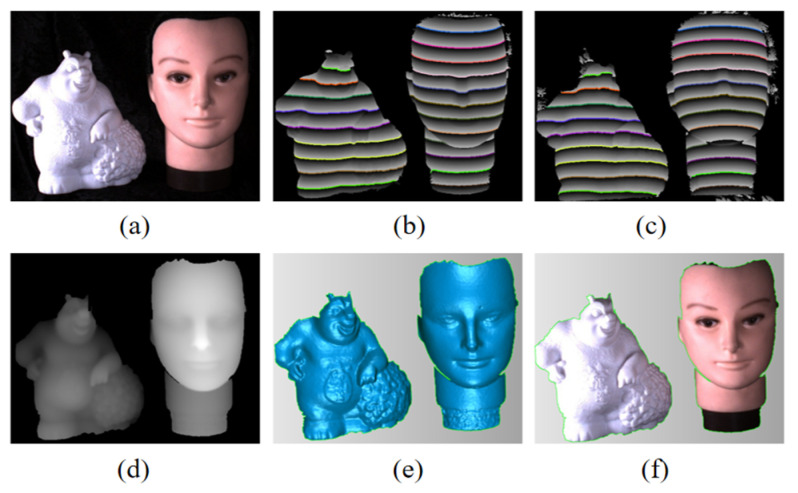
3D reconstruction of multiple objects. (**a**) Photograph. (**b**,**c**) Wrapped phase with matched POLs. (**d**) Disparity map. (**e**) Reconstruction result. (**f**) Textured reconstruction result.

Figure 14 demonstrates the ability of the proposed method to handle spatially separated objects—a key advantage over SPU methods. A 3D face model and a toy bear were placed separately in the field of view. The POLs of the two objects were matched independently, and both objects were reconstructed accurately. This confirms that the proposed method avoids the “island” problem inherent in SPU algorithms.

The experimental results validate the effectiveness of the proposed method. All POL pairs were successfully identified through speckle-based pre-matching, and accurate local matching was subsequently achieved using the wrapped phase information. Notably, the proposed approach achieves performance comparable to that of traditional TPU methods while significantly reducing the number of required patterns (5 vs. 9–12). By eliminating the need for global phase unwrapping, it also overcomes the “island” problem inherent in SPU algorithms, enabling the robust measurement of multiple, spatially isolated objects.

### 3.3. Quantitative Analysis

To quantitatively evaluate the accuracy and efficiency of our method, we performed a comparative analysis using a high-precision optical flat as the reference object. The experimental setup and system calibration were identical to those described in Section 3.1.

**Figure 15:**
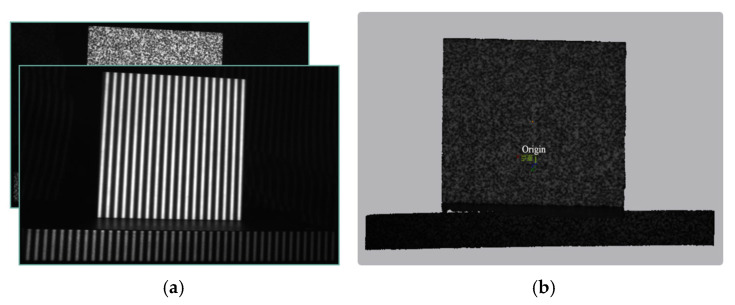

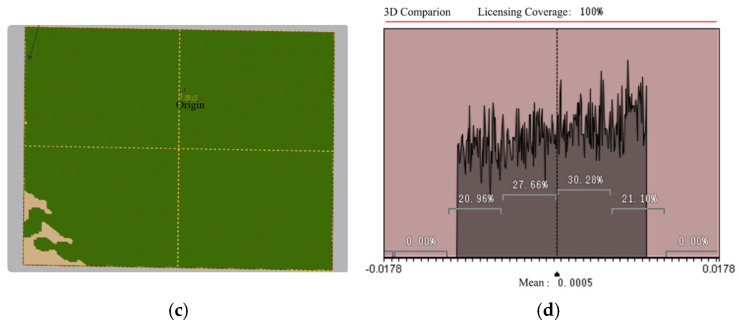
Quantitative accuracy analysis of the proposed method using a high-precision optical flat. (**a**) A subset of the patterns (four fringes and one speckle) projected onto the flat’s surface. (**b**) The reconstructed 3D point cloud, visually demonstrating high planarity. (**c**) A color-coded error map showing the per-point deviation from a best-fit plane calculated via least-squares. The color scale, ranging from −0.2 to +0.2 mm, indicates that most errors are within a narrow band around zero. (**d**) A histogram of the measurement error distribution. The distribution closely approximates a Gaussian curve centered at zero (mean error: 0.003 mm), confirming that the measurement errors are predominantly random and unbiased.

Evaluation Protocol. For each method, we reconstructed the 3D point cloud of the optical flat. A best-fit plane was then computed from the valid (i.e., not masked due to low modulation or saturation) point cloud data using a least-squares algorithm. The measurement error for each point was defined as its orthogonal distance to this fitted plane. We report the mean error (a measure of bias), the standard deviation, and the RMS error. To ensure statistical reliability, all measurements were repeated six times. A representative reconstruction and error analysis for our method are detailed in Figure 15.

Comparison with Baselines. We compared our proposed method against two established techniques: a high-accuracy twelve-step TPU method [37] and a four-step quality-guided SPU method [40]. The quantitative results are summarized in Table 4.

Our method achieves an RMS error of 0.058 mm using only five patterns, which is highly comparable to the 0.048 mm RMS error of the 12-pattern TPU method. This demonstrates a significant improvement in data acquisition efficiency, achieving a 58% reduction in the number of projected patterns without a substantial compromise in accuracy.

Furthermore, this efficiency translates to a higher potential measurement speed. For our system, the total measurement time is dominated by data acquisition, not data processing, as the computational overhead for all compared methods is significantly less than the time required to project and capture the patterns. Therefore, the number of required patterns becomes the critical factor for overall speed. Our 5-pattern method can thus achieve a potential frame rate of 32 FPS, which is about three times faster than the 13 FPS of a typical 12-pattern TPU.

While a single-shot method like SPU offers a similar theoretical capture speed, its accuracy on the optical flat is lower (0.076 mm RMS error). More importantly, as demonstrated in our experiments with complex scenes (Figure 12 and Figure 13), SPU is susceptible to error propagation and fails on spatially isolated objects. In contrast, our proposed method maintains robustness and completeness comparable to TPU in such challenging scenarios, offering a superior balance of speed, accuracy, and reliability.

In summary, the quantitative results validate that our speckle-assisted POL approach strikes an effective balance, delivering accuracy nearly on par with the gold-standard TPU while offering a significant advantage in measurement speed and robustness over SPU.

## 4. Discussion

Advantages. The proposed method offers three key advantages: (1) it requires only five projected patterns, compared to 9–12+ for TPU, enabling faster acquisition; (2) it avoids phase unwrapping entirely, eliminating error propagation and the island problem of SPU; and (3) its fine matching on the wrapped phase achieves sub-pixel accuracy comparable to that of TPU.

Limitations. The method relies on the successful extraction and matching of POLs, which requires sufficient fringe quality (adequate modulation) across the field of view. For objects with very steep surfaces or highly specular reflections, the fringe quality may be degraded, leading to gaps in the POL map. Additionally, the current implementation assumes a single, fixed fringe frequency; incorporating adaptive fringe frequency selection could improve performance for objects with large depth variations. Beyond these, several practical limitations remain:

1. Low-phase SNR or weak modulation: Severe noise, projector/camera defocus, or radiometric nonlinearity (e.g., gamma) can corrupt the wrapped phase, causing missed or fragmented POLs and degraded refinement. Future work could explore advanced denoising filters or high-dynamic-range techniques to improve phase quality in challenging conditions.
2. Thin structures and heavy occlusions: Very thin features or strong occlusions may produce short, broken POL fragments that fail length filtering or cannot be matched reliably. For such cases, multi-scale correlation strategies could be investigated to enhance matching reliability.
3. Challenging materials: Strong specularities, translucency, or very low albedo can bias phase estimation and reduce speckle-correlation reliability. Integrating polarization imaging or photometric stereo techniques could potentially address measurement failures on specular or low-albedo surfaces.

Comparison with embedded approaches. In contrast to methods that embed speckle information directly into fringe patterns [30,31], our approach projects them independently. This preserves the fringe modulation and phase quality, as the speckle pattern does not interfere with the phase calculation. The trade-off is that an additional projection (the speckle pattern) is required, resulting in five patterns instead of four.

## 5. Conclusions

In this paper, a novel phase-matching method based on POL is proposed for binocular-structured light 3D shape measurement. The method requires only five projected patterns (four phase-shifting fringes and one binary speckle) and eliminates the need for phase unwrapping. POLs, formally defined as connected curves of PTPs in the wrapped phase map, provide robust sparse matching cues when combined with speckle-based ZNCC correlation. Dense sub-pixel disparity is then obtained through local fine matching directly on the wrapped phase within each fringe period.

Future work will focus on several areas: (1) extending the method to dynamic scenes using high-speed cameras and three-step phase shifting (requiring only 4 patterns); (2) developing adaptive fringe frequency selection for objects with a large depth range; and (3) integrating GPU acceleration for real-time performance.

## Figures and Tables

**Table 1:** Comparison of representative 3D shape measurement methods.

Method	Patterns	Phase Unwrapping	Island Handling	Sub-Pixel	Accuracy
TPU (multi-freq.) [24,25,26]	9–12+	Required (temporal)	Yes	Yes	High
SPU [21,22,23]	3–4	Required (spatial)	No	Yes	Medium–High
Speckle DIC [18,19,20]	1	Not needed	Yes	Limited	Medium
Composite fringe+speckle [30,31]	4	Required or LUT	Partial	Yes	Medium–High
Deep learning [32,33,34]	1–4	Learned	Yes	Yes	Medium–High
Proposed (POL)	5	Not needed	Yes	Yes	High

**Table 2:** Similarity values between L1 and each right POL.

Right POLs	R_0_	R_1_	R_2_	R_3_	R_4_	R_5_	R_6_	R_7_	R_8_	R_9_	R_10_
ZNCC Score	0.22	0.86	0.11	0.16	0.17	0.23	0.17	0.06	0.08	0.13	0.16

**Table 3:** Stereo matching results of all left-view POLs.

Left POLs	L_0_	L_1_	L_2_	L_3_	L_4_	L_5_	L_6_	L_7_	L_8_	L_9_	L_10_	L_11_
Matched Right POLs	0	1	2	3	4	5	6	7	9	10	Null	Null
ZNCC Score	0.84	0.86	0.89	0.86	0.82	0.85	0.80	0.89	0.65	0.89	0	0

**Table 4:** Quantitative comparison of the optical flat. The results are averaged over six independent measurements. Capture speed is calculated based on the number of patterns required per 3D frame, assuming a maximum camera/projector rate of 160 Hz.

Method	Number of Patterns	Mean Error (mm)	Standard Deviation (mm)	RMS Error (mm)	Capture Speed (FPS)
Proposed Method	5	0.003	0.058	0.058	32
Traditional TPU [37]	12	−0.002	0.048	0.048	13
Traditional SPU [40]	4	0.005	0.076	0.0762	40

## Data Availability

The complete raw data supporting the findings of this study are not publicly available due to ethical and confidentiality restrictions. A de-identified partial dataset sufficient to reproduce the main results can be obtained from the corresponding author upon reasonable request, subject to a data use agreement and, where applicable, institutional approval. Editors and reviewers may request access to these materials under confidentiality for internal evaluation.

## Abbreviations

The following abbreviations are used in this manuscript:

POL	Phase-order line
RMS	Root-mean-square
TPU	Temporal phase unwrapping
SPU	Spatial phase unwrapping
DIC	Digital image correlation
FPP	Fringe projection profilometry
PSP	Phase-shifting profilometry
FTP	Fourier transform profilometry
ZNCC	Zero-Mean Normalized Cross-Correlation
PTP	Phase transition point
